# Healthcare Professionals' Experiences of the Causes of Recurrent Pressure Injuries—A Phenomenographic Study

**DOI:** 10.1111/iwj.71064

**Published:** 2026-09-30

**Authors:** Mari Nööjd, Moustafa Elmasry, Ingrid Steinvall

**Affiliations:** ^1^ Clinical Department of Hand Surgery, Plastic Surgery and Burns in Linköping Region Östergötland Linköping Sweden; ^2^ Department of Biomedical and Clinical Sciences Linköping University Linköping Sweden

**Keywords:** phenomenographic, prevention, recurrent pressure injury, spinal cord injury

## Abstract

There are few previous studies regarding recurrent pressure injuries, most of which investigate surgical outcomes. From the perspective of the healthcare system, recurrent pressure ulcers may indicate shortcomings in quality, as preventive measures have failed not just once, but on several occasions. We aimed to examine how healthcare professionals working with wound care in specialised inpatient care perceived the causes of recurrent pressure injuries in patients with spinal cord injuries. A phenomenographic study using in‐depth interviews was conducted. We identified four categories of the experienced causes of recurrent pressure injuries based on the respondents' subjective accounts: recurrent wounds are due to physical and mental vulnerability in the patient; recurrent wounds are due to a lack of compliance in patients and professionals; recurrent wounds are the result of fruitless endeavours to prevent them; recurrent wounds are due to neglect in healthcare. The causes were described as originating from different structural levels within society. However, preventive measures were primarily perceived as having been assigned at the patient and healthcare staff levels. The elderly were described as particularly vulnerable, whilst care and community interventions declined with increasing age. The participants emphasised that the changes in clinical practice that need to be prioritised primarily concern fundamental principles, in particular person‐centred and inclusive care. Furthermore, a clearer division of responsibilities was highlighted as crucial, as patients in need of multimodal interventions have been found to fall between the cracks in a diagnosis‐based healthcare system.

## Key Points

- This study adds a focus on experienced causes specific to recurrent pressure injuries, an area that has previously been neglected both in research and in clinical practice.
- The causes are described as stemming from various structural levels within society, whilst preventive measures have primarily been assigned at the patient and healthcare staff levels, which has led to ineffective interventions.
- A clearer division of responsibilities is described as crucial, as patients in need of multimodal interventions have been found to fall between the cracks in a diagnosis‐based healthcare system.

## 1 Introduction

The extent to which patients develop pressure injuries is generally considered an indicator of the quality of care [1]. Globally, it is estimated that more than one in ten adults admitted to hospital are affected [2]. Pressure injuries in healthcare are regarded as adverse events and are assessed on the basis of the extent to which they can be prevented [3, 4] It is estimated that up to 95% of all pressure injuries that occur during in‐hospital care could have been prevented through appropriate interventions [3, 5]. Once a pressure injury has developed, measures must be taken to prevent future injuries [6]. Some may argue that recurrent pressure injuries may be a sign of shortcomings in the quality of care, as preventive measures have failed on more than one occasion.

However, interest in factors relevant to recurrent pressure injuries has not been as great as interest in primary pressure injuries in general. This is evident from a scoping review of recurrent pressure injuries that highlights the limited research available. The authors concluded that this has led to a lack of established definitions, diagnostic criteria, and targeted treatment strategies, as well as considerable variations in incidence (5.4% to 73.6%) [7].

Available evidence specific to recurrent pressure injuries comes mainly from the surgical field, where recurrent injuries are the most common complication after reconstructive flap surgery [7, 8, 9]. Patients with spinal cord injuries are a particularly vulnerable group, with a recurrence rate as high as 48%–56% [10, 11, 12].

Risk factors for pressure injuries in general are often classified according to their modifiability. Non‐modifiable risks include age, degree of paralysis, and ulcer location [12]. Modifiable factors mainly consist of clinical findings such as poor nutrition or poorly controlled diabetes, but they also include sociodemographic and lifestyle factors as well as habits [7, 13, 14]. The risk factors for recurrent pressure injuries are largely the same according to a recent review in which low albumin levels were the most frequently reported risk factor, followed by spinal cord injury, advanced age, duration of hospital stay, and wound size [7]. The evidence base for preventive measures is extensive, but interventions such as nutrition and repositioning are described as having low certainty [15, 16], and the use of risk assessment tools has not significantly affected the incidence [6, 17]. The attitudes and knowledge of healthcare staff are highlighted as an important aspect of prevention, with a number of studies that identify shortcomings in this area [4, 18, 19, 20].

Therefore the aim of this study was to examine the causes of recurrent pressure injuries in patients with spinal cord injuries, as experienced and described by professionals who meet this patient group in their clinical work.

To investigate the aim we planned to interview clinicians with experience of patients with recurrent pressure injuries to investigate the following research questions:

- How do healthcare professionals working with wound care in specialised inpatient care perceive the causes of recurrent pressure injuries in patients with spinal cord injuries?
- What are the similarities and differences between the various ways in which the causes of recurrent pressure injuries are described?

## 2 Methodology/Method

Phenomenography is considered a useful approach based on its focus on human experience. It was defined by its pioneer Ference Marton as ‘a research method adapted to map the qualitatively different ways in which people experience, conceptualize, perceive, and understand various aspects of, and phenomena in, the world around them’ [21]. The methodology is based on the assumption of relationality, creating a second‐order perspective, as it is the individual's experiential relationship to a phenomenon that is in focus [22]. The analysis focuses on collective experiences rather than individual ones and can thus provide knowledge that encompasses both variation and range [21, 22, 23, 24].

### 2.1 Participants

A purposeful selection of clinicians was used to capture the diversity in ways of describing and experiencing the phenomenon of recurrent pressure injuries.

Two different departments, Rehabilitation Medicine and Plastic Surgery, at a University Hospital in a high income country that encounter and treat patients with spinal cord injuries and recurrent pressure injuries were selected. The first author contacted potential respondents who met the criteria set out. The focus was directed towards the staff who were actively involved in the patients' clinical wound care. The variation that was considered most important to capture concerned the different care assignments that these two clinics have; lifelong rehabilitation and intervention‐driven complex surgery. Within the different departments, diversity based on gender, profession, and clinical experience (a minimum employment period of 2 years was the limit) was sought (Table 1).

**TABLE 1:** Participants in the study based on department affiliation, gender, occupation, and duration of employment.

Department of Rehabilitation Medicine	Department of Hand and Plastic surgery
2 men 4 women	2 men 6 women
2 physicians 2 nurses 2 nursing assistants	3 surgeons 4 nurses 1 nursing assistant
Duration of employment 5–30 years	Duration of employment 4–20 years

Fourteen individual semi‐structured interviews were conducted between November 2024 and November 2025, which is in line with a general recommendation being 10–30 participants [25].

All respondents participated on a voluntary basis. Verbal and written informed consent was obtained. The study was approved by the National Ethical Review Authority.

### 2.2 Interview Design

Individual, in‐depth interviews with few, open‐ended questions were used [26]. Interviews began by asking respondents to describe their professional background and their experiences of meeting patients with spinal cord injury and recurrent pressure injuries. The interviews then continued with predetermined open‐ended questions, followed by follow‐up questions aimed at confirming, clarifying and deepening understanding (Supporting Information: File S1).

Respondents were asked throughout the interview to illustrate their statements with examples from their everyday clinical practice. During the interview, notes were taken which were used to connect arguments or link statements that came up during the interview with things that had been said earlier.

### 2.3 Reflexivity

Phenomenographic analysis uses an interpretative approach to the data collected [22]. Preconceived notions and personal truths held by those engaged in interpretative analysis must therefore always be acknowledged [26]. The first author has a background as a nurse in plastic surgery and is familiar with the care process from an intervention‐based perspective. The first author conducted the interviews and performed the analysis. The second author acted as a critical reviewer throughout the whole process. To identify any specific perceptions of the first author at the beginning of the study, the second author interviewed the first author. This interview was transcribed but has not been analysed as part of the study's results. Instead, it has served as a basis for critical awareness and personal reflection during the course of the study.

### 2.4 Analysis

The analysis was carried out as an iterative process, according to the 10 steps by Åkerlind (Table 2) [22] and aimed to provide answers to:

- What are the qualitatively different ways in which the phenomenon can be understood?
- What dimensions of variation of the phenomenon are discerned within each way of understanding it?

**TABLE 2:** The 10‐step analysis by Åkerlind.

Familiarising	The interviews were transcribed by the first author and then read through in their entirety several times.
Condensing	Statements based on how respondents described their own experiences of the causes of recurrent pressure injuries, but also based on how respondents described the meaning of the phenomenon in question, were marked in the text. The descriptions were then interpreted based on their context (the transcription as a whole) and meaningful units were formed. The condensed, meaningful excerpts were written down on post‐it notes, which were mixed to form a collective pool of meaning.
Comparing	The collective pool of meaning was then organised and reorganised based on similarities and differences in how perceived causes and underlying factors for the occurrence of recurrent pressure injuries were described.
Grouping	Preliminary categories of descriptions were created based on the common view of causes that emerged from the comparison of the collective pool of meaning.
Delimiting	What linked the categories and what distinguished them, as well as whether the different categories covered all the aspects that had emerged or whether more categories were needed to cover the entire variation was discussed within the research group.
Discerning	What constituted the critical core of each category and what distinguished the different categories from each other was discussed and formulated in terms of dimension of variations.
Articulating	The overall meaning of each category (the whole) and its inherent components (the parts) was formulated and described.
Checking	As interpretations were formulated, they were checked against original and condensed transcripts to ensure their relevance, but also to question their validity.
Relating	An outcome space with increasing complexity in terms of causes of recurrent pressure injuries was created.
Labelling	Categories and dimensions of variation were provided with headings that clearly conveyed their content.

Descriptive categories (C) and what characterises and distinguishes these, in the form of dimension of variation (D), were developed during the analysis. The categories were then related hierarchically based on their complexity resulting in an outcome space.

Complexity is not a matter of finding the highest level of expertise, but rather of finding the description that is most inclusive and expansive [22].

Quotations have been used to illustrate both the respondents' perceptions and the authors' interpretations [27]. The selection process for quotations was based on how well they were considered to illustrate the conceptual standpoint of the categories and thus speak for more respondents than just the one quoted [22].

## 3 Results

We identified four qualitatively distinct categories of the experienced causes of recurrent pressure injuries (C1): Recurrent wounds are due to physical and mental vulnerability in the patient (C2): Recurrent wounds are due to a lack of compliance in patients and professionals (C3): Recurrent wounds are the result of fruitless endeavours to prevent them (C4): Recurrent wounds are due to neglect in healthcare. Each category and its inherent dimensions of variation (D 1–8) are described below. The first paragraph of each category is a summary.

### 3.1 **Category 1**—Physical and Mental Vulnerability—The Medical Perspective

- D1 Secondary complications
- D2 The ageing patient

In category 1 (C1), respondents described the perceived causes of recurrent pressure ulcers as being linked to the patient's physical and mental abilities and the inevitable ageing of the body. This perspective was expressed mainly in physiological terms.

Trigger factors were perceived to be related to the consequences of spinal cord injury, such as paralysis, loss of sensation or muscle atrophy. Well‐known risk factors for pressure injuries, such as weight change, poor nutrition, and abnormal clinical parameters in terms of haemoglobin and albumin, were also mentioned here, as well as previous wounds and operations that were considered to result in more sensitive and fragile skin.

Participant (P1): The patient is paralysed, so you think that's how it will be, that it's difficult for that patient, that they can't feel if it's coming back,

(P10): Well, there are, of course, risk factors such as the type of injury, mobility, skin fragility, weight, both being underweight and overweight—sweating, (…) and incontinence. I mean it's very difficult to pinpoint one thing.

Age and ageing were described as a specific risk factor, as this can affect mental capacity and contribute to general weakness, both due to an increased number of comorbidities and age‐related changes in the body's constitution. The combination of living with a spinal cord injury and ageing was perceived as something that greatly increased the risk of recurrent pressure injuries, which was contrasted with the experience that access to good medical care decreased with increasing age.

(P10): Everyone knows that we all get older, but from a medical and physical standpoint, a body with a spinal cord injury ages faster—you wear yourself down and lose muscle mass (…) so it might be that they're sitting on the same cushion they were sitting on 20 years ago, and that just doesn't work, does it?

Other aspects of ageing that were highlighted included the fact that personal assistance is only available to people under the age of 65, as well as examples of how selection for surgery and access to highly specialised care for spinal cord injuries were based on factors such as age, the absence of recurrent injuries and comorbidities.

(P11): We select which patients are to undergo surgery based, among other things, on how the pressure ulcer has developed; if, in addition, we know that the patient is elderly and lives in a care home where he is not repositioned properly, then he is not a particularly suitable candidate for surgery.

(P9): The highly specialised units screen patients, and if a patient is not accepted—for example, due to age, limited prognosis, or comorbidities (…) the patient may end up directly in short‐term housing, where the conditions for caring for a complex spinal cord injury in an older person are less favourable.

### 3.2 **Category 2**—Lack of Compliance—What Needs To Be Done Is Not Being Done

- D3: Motivation deficit
- D4: Knowledge gaps

In category 2 (C2), respondents provided a more complex picture of why recurrent pressure injuries occur. It was recognised that recurrent pressure injuries are a secondary complication following spinal cord injury. However, this was not considered to be the complete explanation, as some people with spinal cord injury never develop pressure injuries, while others develop them repeatedly. The causes highlighted in C2 instead referred to situations in which compliance with preventive measures had been neglected or forgotten, and these cases of non‐compliance were attributed to both patients and professionals.

Respondents described the importance of how preventive measures in the form of life‐affecting restrictions needed to be accompanied by motivational and supportive measures in order to be successful. Despite this, it was rarely offered, which one participant *(P5)* explained as follows: ‘It becomes very focused on a single wound, not on the whole picture; that's what we're missing’.

Patients who had relatives had to rely on them, while others lived without care, support, and compassion, which was considered to affect the ability to maintain necessary motivation over a longer period.

(P7): He had been paralysed for 20 years or so (…) and had undergone many operations on his bottom, so he had a lot of scars and things like that (C1) (…) he understood, but he couldn't cope with always being so responsible. (…) And we said, now you have to do this. Yes, and then it worked for two days and then it was like nothing more, and so all of that probably made life so difficult sometimes. So, he couldn't manage to fix this (C2).

The reasons for poor compliance were also linked to healthcare professionals who lacked the necessary knowledge, worked with insufficient resources or were simply unaware of their responsibilities. Well‐established routines were considered fundamental to maintaining good compliance, and this was described as lacking, both in terms of the management of recurrent pressure injuries and in terms of who is responsible for what, which led to the prevailing perception that there was always someone else to blame when pressure injuries occurred.

(P1): We do have checklists and procedures we follow and all that, but then things don't always turn out as planned anyway—that's just how it is—but I sort of think that often, they aren't really followed properly after all. And then there's other information, and things turned out differently, so it all gets a bit messy sometimes, in a way. And then there are quite a lot of people involved with the same patient. Who's said what? Has anyone said anything at all?

### 3.3 **Category 3**—Fruitless Endeavours—The Lack of a Holistic Approach

- D5: Inadequate health literacy
- D6: Insufficient person‐centredness

This category (C3) describes the causes from a perspective that not only encompasses characteristics and compliance (C1, C2) but also highlights how measures taken are wasted if they are not adapted to the individual patient and evaluated. The lack of a holistic perspective was reflected in the respondents' descriptions of routine‐driven care and its influence on knowledge exchange.

The reason for recurrent pressure injuries was described as a perception that too much reliance was placed on general standardised information when it comes to preventing pressure injuries. That the same information tended to be repeated also, even when it had not been shown to lead to any desired result.

(P8) We treat everyone equally. I think so if you inform them. But as I said, then you realise that yes, people are different; they take different things seriously.

The shortcomings of this approach were explained by the fact that people with spinal cord injuries are not a homogeneous patient group, but differ in terms of personality, previous experiences and future plans. Respondents stated that important knowledge about risks and preventive measures must be so well integrated into the patient's consciousness that it can be retrieved and used independently in a variety of situations in life. The fact that this is not currently the case was illustrated by an experience that pressure injuries often arise due to random causes resulting from deviations from the standard routines. *(P7)*: ‘(…) then they end up getting them anyway whilst they're in the sauna, or someone sat on a hot bonnet—or on an aeroplane’.

The information that patients received was described as based on what healthcare providers wanted to convey. The consequences of this top‐down perspective, where information replaces collaboration, were described as the patients, rather than being invited to participate, were being left out of decisions that affected their own lives. Truly difficult issues were also at risk of never even being brought up for discussion. (P4): ‘You really must find out—by talking to the patient (…) but I don't think everyone does that. Not everyone takes the time’. The opinion that the patient is the most important person when it comes to successfully preventing pressure injuries was expressed in all interviews. This was further explained by the fact that people with spinal cord injuries often act as supervisors and trainers of home care staff or personal assistants in everyday life, whose job it is to prevent secondary complications. Patients thus constitute the continuous link to specialist care and whether recurrent pressure injuries can be avoided was described as a causal relationship dependent upon how knowledgeable and educated these patients are.

(P10): I don't think any of these patients are unaware, because they have been told repeatedly, but sometimes it gets lost and then it doesn't help to repeat (…) what we must do, because in a sense the patient is alone with their body and their life, is to get the individual to reflect and identify and teach the patient to identify risks themselves, a more CBT‐oriented approach to behaviour and thereby find strategies that are individual. We need to consider whether we are doing things the right way. We have an exaggerated belief that information leads to behavioural change, and there is very little evidence to support this (C3).

### 3.4 **Category 4**—A Neglected Patient Group—A Fragmented Healthcare System

- D7: A fragmented health care.
- D8: Shortcomings in prioritisation

Category 4 (C4) describes the causes as a chain of events that starts in the individual patient's needs and how well these are met, which is determined by priorities in healthcare and society. The causes within this category are attributable neither to the patient alone nor to individual caregivers, as the inability to implement successful preventive measures is described largely due to a system in which those most in need of prioritisation were seen to be offered the least resources.

The category describes how the development towards a specialised healthcare system has affected patient groups with multifactorial needs and led to them being neglected both organisationally and in terms of care. Patients with recurrent pressure injuries have many health care contacts, without anyone feeling full responsibility. The causes are described as arising from gaps in knowledge, responsibility and information between these different contacts. *(P12): ‘When they come to us, you might think that certain things should already have been sorted out, but you don't really know’*.

The neglect of this patient group was perceived to be due to priorities. Treatment of recurring pressure injuries is generally not considered a priority task for specialised inpatient care. The needs are not considered to fall fully within the scope of any of the included clinics' operational mandates either. ‘We *offer rehabilitation, not wound care—that's not making optimal use of our beds’ (P14)*.

In the absence of the possibility to provide inpatient care, healthcare personnel were recommended to try to anticipate needs and prevent recurring wounds by writing medical assessments and certificates. If the necessary competence was lacking or time constraints prevented a full understanding of the patient's situation, these assessments risked leading to inadequate interventions. Experience also showed that assessments were increasingly questioned by the Social Insurance Agency, and that, for example, the need for motivating measures, did not justify interventions in the form of personal assistance. One participant summarised the chain of events as follows, which reflected all four categories in the outcome space.

(P9): We have an elderly person with complicating risk factors, a comorbidity including diabetes, which further impairs both sensitivity and healing ability (C1), (…) and at the same time, the system is not functioning as we would like, and the patient is not receiving the support they need (C2). (…) there is a lack of knowledge within the system, or the knowledge is not being utilised to the extent that it should be (C3). (…) It is also us who are not doing enough; it is the state that has made a change at the system level without being fully aware of the consequences, but there is also a lack of ability to take responsibility on the part of the patient and on the part of people, colleagues and individuals close to the patient. So I don't think you can point the finger at a single person or a single function; as is so often the case when things don't go as planned, there are several factors that are not working as they should (C4).

### 3.5 Outcome Space

The outcome space is a representation of the logical connection between the subjective perceptions of the causes of recurrent pressure injuries. The outcome space is hierarchically structured and represents the study population's collective experience‐based knowledge based on its increasing complexity and the degree of inclusion involved (Figure 1).

**FIGURE 1:**
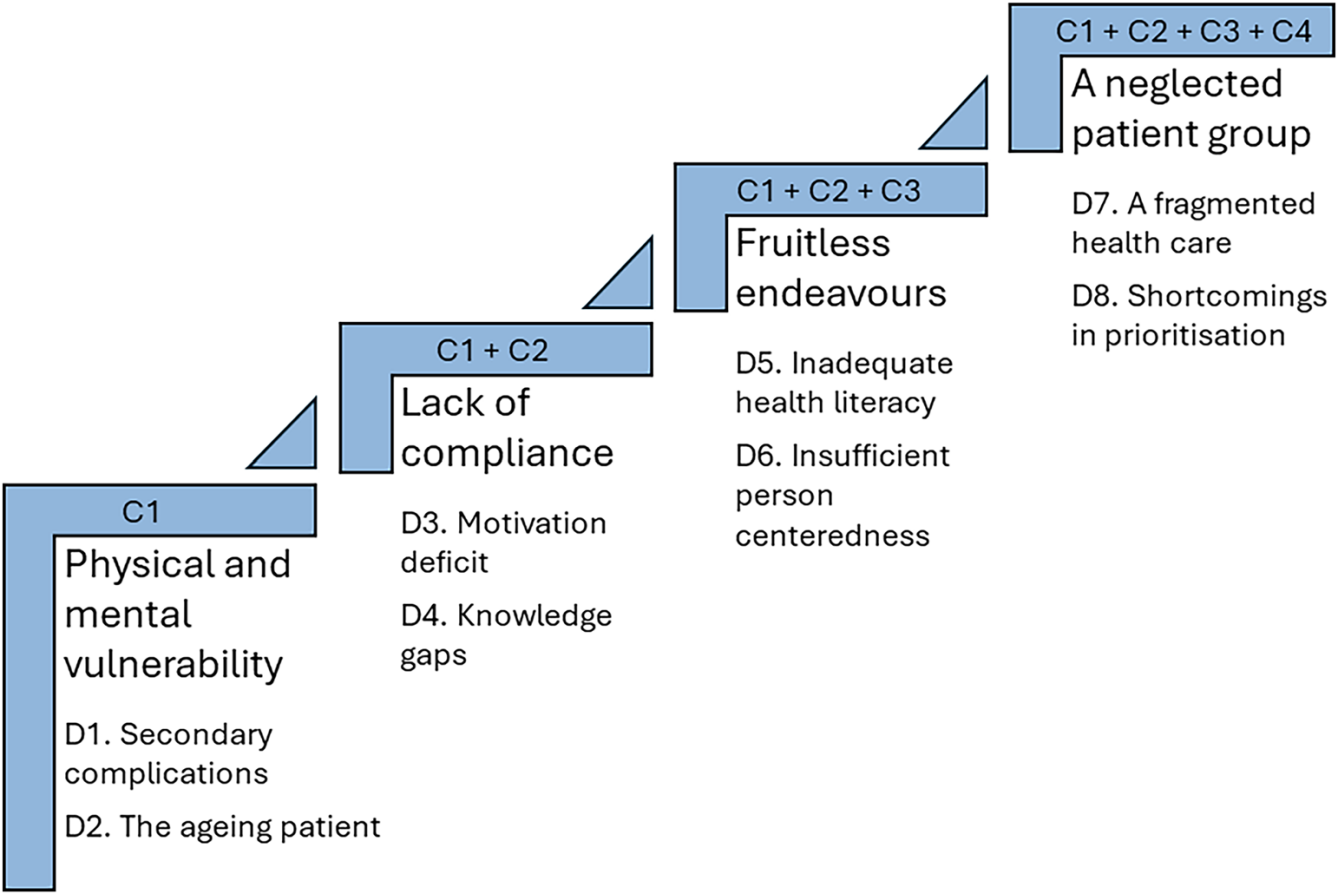
Outcome space of the experienced knowledge of the causes of recurrent pressure injuries. The outcome space is presented in the form of a staircase in which each step of the staircase represents an increased complexity (C1‐4). Each step relates to the next by including previous conceptions both in the form of categories (C) and dimensions of variation (D). The highest step thus encompasses all categories as well as dimensions of variation and is therefore interpreted as the most complex category, drawn from this sample at the time of the interviews.

## 4 Discussion

### 4.1 Statement of Principal Findings

The purpose of this study was to gain a better understanding of the causes of recurrent pressure injuries in patients with spinal cord injuries, as experienced and described by professionals who meet this patient group in their clinical work. We used a phenomenographic approach which revealed a complexity comprising several layers of explanatory factors and highlighted that no one is specifically assigned to this area of care in the long term. What is new about this study is not the individual risk factors themselves [7], but the way in which they are brought together to provide a deeper understanding of the challenges involved in preventing recurrent pressure injuries.

Categories 1 and 2 largely address the characteristics of the patients and their closest caregivers. Added to this is ageing, which was described as a factor in its own right, not least because age can act as a barrier to accessing the most specialised care but also because there is an age limit for personal assistance. Previous research has provided strong evidence that age is a significant risk factor for recurrent pressure injuries [28]. As the skin becomes more fragile with age and thus more sensitive to moisture, friction and trauma, and as metabolism changes and circulation deteriorates, more multifaceted interventions are needed [7]. Inadequate care due to age, leading to adverse health effects, has been reported from many areas across the world [29, 30].

To avoid recurring pressure injuries, respondents emphasise the importance of making patients aware of risk situations and risk behaviours, to educate them. What distinguishes category 2 from category 3 is the approach itself, where category 2 largely accepts the existing approach with leaflets and general information, while category 3 questions and criticises it for being simplistic and ineffective. There is evidence that knowledge plays an important role in the prevention of pressure injuries [31]. However, there is also evidence to suggest that information alone rarely leads to change [32].

Category 3 describes that the causes are due to medical factors or poor compliance, but that this actually originates in core values, or working methods that have not been designed or adapted to meet the needs of this patient group. This is illustrated by descriptions of a standardised care that fails to put the individual at the centre, leading to a lack of cooperation and ineffective, inflexible solutions. It has been suggested that moving away from this paternalistic approach and instead adopting consultations based on concordance, where the focus is on the patient's experiences, knowledge and wishes, has the potential to lead to greater adherence and compliance [33, 34].

The need for a more holistic approach is in line with reported needs of patients with spinal cord injuries, which suggests that treatment must be tailored to patients' individual wishes and abilities, and that greater consideration must be given to the social consequences of avoiding or healing pressure injuries [35]. Achieving this by building and maintaining partnerships and providing support for self‐management are some of the measures supported by research [36, 37].

Category 4 raises the focus to a level that includes both organisational, political, and economic decisions regarding priorities and healthcare systems. Shortcomings at this level then go back and forth through the structures, via healthcare facilities and healthcare personnel, and ultimately affect the patient in the form of recurrent pressure injuries. The causes are largely attributed to a healthcare system in which each diagnosis is handled separately and patients with multifactorial needs are deprioritised, which leads to unclear division of responsibility, insufficient knowledge, and a lack of resources. The existence of this fragmented, silo‐based care model has been noted to cause difficulties even for other patients with multimodal problems [38, 39]. It has been described as a lack of competence, continuity, and priority, which leads to wounds worsening and care processes being prolonged [40].

Having a spinal cord injury means living at high risk for pressure injuries [41]. There is, however, a danger in normalising the occurrence of pressure injuries within a specific group. Learned helplessness is a condition in which individuals, both patients and professionals, after repeated exposure to uncontrollable negative experiences, lose their motivation, become passive and cease their attempts to change [42, 43, 44]. Patients with recurrent wounds have expressed less confidence in their own bodies and abilities to avoid pressure injuries than patients who suffered from them for the first time [45]. As the preventive measures taken are mainly focused on influencing patient‐related factors, and thus have little impact on the overall structures, this provides a possible answer to the study's initial question of why recurrent pressure injuries continue to occur. This is similar to the framework developed by Balzer et al. who emphasise that factors such as the organisation of care processes have as great an impact on the risks as patients and healthcare personnel [6]. Cesca et al. point out that the challenge of preventing and managing pressure injuries lies with the patient, but also with healthcare professionals and the healthcare system [46].

### 4.2 Implications for Policy, Practice and Research

The findings of this study highlight an inequality in care that needs to be addressed in a way that reflects the complexity of healthcare organisation and delivery at different levels. The findings may be of interest to decision‐makers in society, as unsuccessful healthcare interventions risk leading to even greater healthcare needs and higher costs. The findings can also be applied in clinical practice to help patients and healthcare professionals understand why current preventive measures are not working as effectively as they could, and what needs to be done to make further progress. The study is also expected to inspire further research to explore how recurrent pressure injuries can be prevented through a more individualised approach.

### 4.3 Strengths and Limitations

The findings in this study are based on two different departments within the same hospital. The outcome space indicates that the extent of the problem is broader. The aim of the study, however, was to focus on the professions that are actively involved in wound care. It would be interesting to repeat the study with respondents from different levels of care, other professions, or those at a decision‐making level, to see if it would enlarge the outcome space.

## 5 Conclusion

The causes of recurrent pressure injuries in patients with spinal cord injuries are described by healthcare professionals as originating from several structural levels within society. However, preventive measures are primarily perceived as being assigned at the patient and healthcare staff levels, leading to fruitless results.

The elderly were described as particularly vulnerable through all levels of complexity, while care and community interventions were described as decreasing with increasing age, which is the very meaning of the inverse care law, and leads to substandard care among the elderly.

The areas of clinical practice most in need of change relate to the core values of care and a clear division of responsibilities. More individualised, inclusive, and person‐centred care needs to be prioritised. This is particularly important given that patients with a need for multimodal interventions have been shown to fall between the cracks in a diagnosis‐based healthcare system.

## Funding

The authors have nothing to report.

## Ethics Statement

The study was preceded by the Swedish Ethical Review Authority (DNR 2021–03613, application for amendment DNR 2024–06018‐02).

## Consent

Written and oral information about study participation was provided to the respondents and written informed consent was obtained before the interviews took place.

## Conflicts of Interest

The authors declare no conflicts of interest.

## Supporting information


**File S1:** Interview guide.

## Data Availability

The data that support the findings of this study are available from the corresponding author upon reasonable request.
