# Supplementary material for: Healthcare Professionals' Experiences of the Causes of Recurrent Pressure Injuries—A Phenomenographic Study
Source: Int Wound J. 2026 Sep 30;23(10):e71064. doi: 10.1111/iwj.71064 (PMC13625209; doi:10.1111/iwj.71064)
Supplement: Supplementary file 1 [file IWJ-23-e71064-s001.docx]

**Supplemental file 1. Interview guide.**

**Introduction**

- Can you tell me what you do for a living and a little about your background?
- Could you describe your experience of meeting patients with recurrent pressure injuries?

**Main questions**

Can you try to think back to a specific patient with recurrent pressure injuries that you have encountered

- Can you tell me about how and why you met?
- What were your thoughts about this patient being affected?
- Can you give an example of something you did at this specific meeting that you think matters when it comes to the risk of future wounds?
- Are there things in this meeting, or in other patient meetings, where you feel/wish you could have done more?
- Do you believe that recurrent pressure injuries are preventable?

**Probes**

- Can you elaborate on why you think this?
- Can you tell me a little more?
- Can you give an example?
- What did you do then?
- Why did you do that?

**Conclusion**

- Is there anything else you would like to add?
